# Assessing sustainment of health worker outcomes beyond program end: Evaluation results from an infant and young child feeding intervention in Bangladesh

**DOI:** 10.3389/frhs.2022.1005986

**Published:** 2023-01-09

**Authors:** Corrina Moucheraud, Adrienne Epstein, Haribondhu Sarma, Sunny S. Kim, Phuong Hong Nguyen, Mahfuzur Rahman, Md. Tariquijaman, Jeffrey Glenn, Denise D. Payán, Purnima Menon, Thomas J. Bossert

**Affiliations:** ^1^ Department of Health Policy and Management, Fielding School of Public Health, University of California, Los Angeles, Los Angeles, CA, United States; ^2^ Department of Vector Biology, Liverpool School of Tropical Medicine, Liverpool, United Kingdom; ^3^ National Centre for Epidemiology and Population Health, Australian National University, Canberra, ACT, Australia; ^4^ International Food Policy Research Institute, Washington, DC, United States; ^5^ International Centre for Diarrhoeal Disease Research (ICDDR), Dhaka, Bangladesh; ^6^ Department of Public Health, Brigham Young University, Provo, UT, United States; ^7^ Department of Health, Society, and Behavior, University of California, Irvine, CA, United States; ^8^ International Food Policy Research Institute, New Delhi, India; ^9^ School of Public Health, Harvard University, Boston, MA, United States

**Keywords:** implementation science, sustainability, public health, global health, Bangladesh, infant and young child feeding (IYCF)

## Abstract

**Introduction:**

Alive and Thrive (A&T) implemented infant and young child feeding (IYCF) interventions in Bangladesh. We examine the sustained impacts on health workers' IYCF knowledge, service delivery, job satisfaction, and job readiness three years after the program's conclusion.

**Methods:**

We use data from a cluster-randomized controlled trial design, including repeated cross-sectional surveys with health workers in 2010 (baseline, *n* = 290), 2014 (endline, *n* = 511) and 2017 (post-endline, *n* = 600). Health workers in 10 sub-districts were trained and incentivized to deliver intensified IYCF counseling, and participated in social mobilization activities, while health workers in 10 comparison sub-districts delivered standard counseling activities. Accompanying mass media and policy change activities occurred at the national level. The primary outcome is quality of IYCF service delivery (number of IYCF messages reportedly communicated during counseling); intermediate outcomes are IYCF knowledge, job satisfaction, and job readiness. We also assess the role of hypothesized modifiers of program sustainment, i.e. activities of the program: comprehensiveness of refresher trainings and receipt of financial incentives. Multivariable difference-in-difference linear regression models, including worker characteristic covariates and adjusted for clustering at the survey sampling level, are used to compare differences between groups (intervention vs. comparison areas) and over time (baseline, endline, post-endline).

**Results:**

At endline, health workers in intervention areas discussed significantly more IYCF topics than those in comparison areas (4.9 vs. 4.0 topics, *p* < 0.001), but levels decreased and the post-endline gap was no longer significant (4.0 vs. 3.3 topics, *p* = 0.067). Comprehensive refresher trainings were protective against deterioration in service delivery. Between baseline and endline, the intervention increased health workers' knowledge (3.5-point increase in knowledge scores in intervention areas, vs. 1.5-point increase in comparison areas, *p* < 0.0001); and this improvement persisted to post-endline, suggesting a sustained program effect on knowledge. Job satisfaction and readiness both saw improvements among workers in intervention areas during the project period (baseline to endline) but regressed to a similar level as comparison areas by post-endline.

**Discussion:**

Our study showed sustained impact of IYCF interventions on health workers' knowledge, but not job satisfaction or job readiness—and, critically, no sustained program effect on service delivery. Programs of limited duration may seek to assess the status of and invest in protective factors identified in this study (e.g., refresher trainings) to encourage sustained impact of improved service delivery. Studies should also prioritize collecting post-endline data to empirically test and refine concepts of sustainment.

## 1. Introduction

Optimal breastfeeding and complementary feeding (defined as timely and adequate introduction of appropriate foods to an infant's diet in addition to breastmilk) strongly influence nutrition, growth and health outcomes in children (1, 2). Poor early-life nutrition contributes to malnutrition-related conditions (e.g., stunting) (3, 4) and delayed child development (5), and may cause up to an estimated 45% of all child deaths worldwide (6). Although some interventions to improve infant and young child feeding (IYCF)—i.e., optimal breastfeeding and complementary feeding—have achieved improvements, they have struggled during scale-up and sustainment (7, 8).

Sustaining components of effective interventions and program activities is essential for maintaining and supporting improvements in IYCF practices. Whether and how interventions are integrated into ongoing practices and institutions, for example through building capacity, can support sustainable implementation (6). However, there are many challenges to sustaining momentum or effective results with unpredictable funding resources that are largely donor driven, like limited multisectoral coordination and inadequate personal capacity (including high employee turnover) (9).

Sustainment can be conceptualized as ongoing activities that continue to result in improved outcomes (10). Sustainability of donor-funded programs is a crucial but understudied issue. As international organizations and donors increasingly wish to transition implementation responsibilities to recipient countries, and in some cases, ultimately phase out funding (11–13), it is imperative to better understand factors that enable (or hinder) lasting impacts from programs (10, 14–22). Data are lacking on the dynamics of outcomes affected by donor-funded programs after funding has ceased, and on factors that contribute to program sustainability (23, 24). Therefore, to advance the field of implementation science, research is needed evaluating longer-term outcomes of sustainability (e.g., sustainment) (25).

Alive & Thrive (A&T) supported nutrition interventions to improve maternal nutrition and infant and young child feeding practices in several countries including Burkina Faso, Ethiopia, India, Nigeria and Vietnam. In Bangladesh, A&T was implemented from 2009 to 2014 as a demonstration project of an at-scale model for achieving IYCF improvements (see ***Program Description***, below). Findings from impact evaluation showed that A&T was associated with improved IYCF knowledge and behaviors: health workers in intervention areas had significantly greater improvements in IYCF knowledge and job motivation during the program period (2010 to 2014) relative to workers in comparison areas (26). A post-endline evaluation (conducted in 2017) of health workers in these same areas similarly found significantly better IYCF knowledge and job satisfaction in intervention vs. comparison areas (14, 27). While there is evidence that certain outcomes persisted beyond the end of the project period, data from baseline, endline, and post-endline have not been linked to estimate the degree of sustainment. Each evaluation effort deliberately aligned study samples and survey instruments, so it is possible to analyze the degree to which any intervention effects observed from 2009 to 2014 persisted until 2017.

In this paper, we investigate the presence and magnitude of “voltage drop”—i.e., attenuation of benefits over time (28)—after the A&T program in Bangladesh ended in 2014, i.e., the extent to which improvements in health worker outcomes in intervention areas were sustained or returned to the same level as in the comparison areas. This is an important area of study since capacity and resource limitations were identified as potential issues that could curtail scale-up and long-term improvements for A&T (29). Is there evidence of sustained differences in outcomes (quality of IYCF service delivery, IYCF knowledge, job satisfaction and job readiness) among health workers in intervention areas, vs. those in comparison areas, after the A&T initiative ended in Bangladesh? We also examine whether changes in these outcomes post-endline were differentially affected by program activities i.e., refresher training and receipt of financial incentives.

## 2. Materials and methods

### 2.1. Program description

Alive & Thrive (A&T) is an initiative supported by the Bill and Melinda Gates Foundation that aimed to demonstrate at-scale improvements in IYCF behaviors in Bangladesh, Ethiopia and Vietnam during Phase 1 from 2009 to 2014 [see detailed descriptions (26, 29, 30)]. Specific intervention details varied in each country but had a common core that included: interpersonal counseling, mass media, community mobilization, and policy advocacy activities– all bolstered by partnerships and strategic use of data (31, 32). In Bangladesh specifically, the A&T initiative included interpersonal communication (IYCF counseling with pregnant women and mothers of young children) and community mobilization (local meetings with stakeholders and village theater performances); these components were delivered by BRAC, a large non-governmental organization with a network of community-based volunteers, in areas assigned to A&T interventions. Mass media (television commercials and radio stories about IYCF) and policy advocacy (dissemination and the creation of a National IYCF Alliance) were also conducted country-wide across intervention and comparison A&T areas.

**Figure 1:**
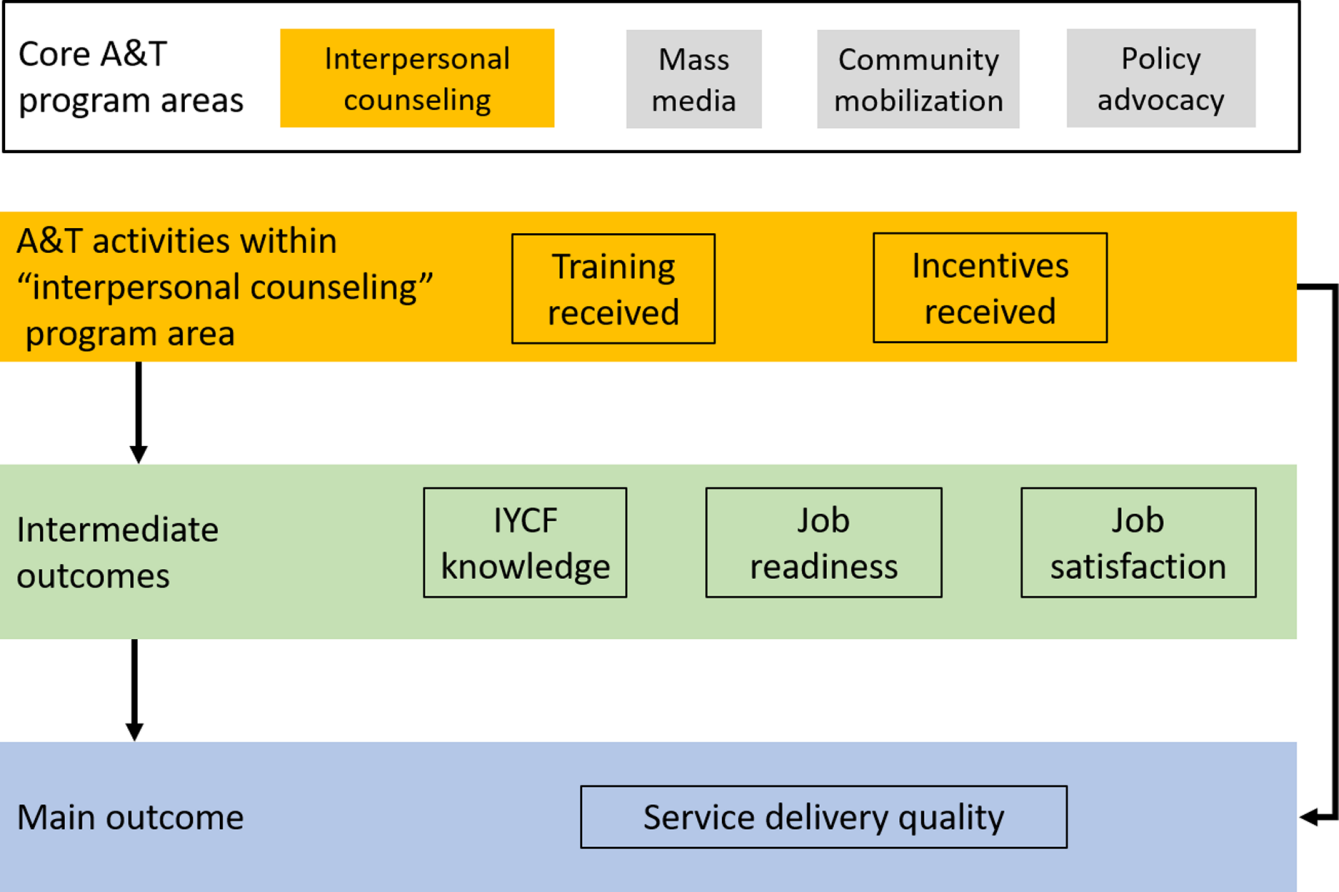
Conceptual framework of the hypothesized relationship between Alive & Thrive Phase 1 program areas and activities, and outcomes among health workers (intermediate and main outcomes).

Here, we focus specifically on the interpersonal communication (IYCF counseling) and community mobilization components of the A&T program in Bangladesh. The unit of analysis are frontline health workers who deliver at-home health services to pregnant women and mothers of young children. We conceptualize the main outcome of interest as the quality of IYCF service delivery, operationalized as the quantity of IYCF topics discussed in counseling sessions. Quality of service delivery is imperative as it is associated with downstream improved IYCF outcomes among clients (33). We hypothesize that this is influenced by intermediate outcomes of IYCF knowledge, job readiness and job satisfaction—and that, in turn, these can be impacted by program activities of training and incentives (Figure 1).

### 2.2. Study design

A&T Phase 1 was implemented using a cluster-randomized control trial design, and impact evaluation data were collected *via* cross-sectional surveys conducted in 2010 and 2014. There was a random selection of 20 rural upazillas (sub-districts) for the trial: 10 received the intervention A&T Phase 1 package of activities (the intervention areas) and 10 continued to implement standard care by BRAC frontline workers (comparison areas). These 20 upazillas were selected from among 357 upazillas in the participating 5 (of 7) divisions of Bangladesh. More information about the selection and randomization process has been previously published (14, 26, 34).

### 2.3. Data collection

Across the 20 participating upazillas in Bangladesh, 200 villages were randomly selected for data collection (from 3581 villages total); these 200 villages comprised the sampling frame for all three rounds of data collection. All health workers in these villages were surveyed in 2010 (baseline) and 2014 (endline). In 2017 (post-endline), these upazillas were revisited and health workers were randomly sampled using BRAC's rosters (ranging from 2 to 9, depending on the number available). To be eligible for participation in 2017, the health worker must have had a planned household visit within 2 days of initial contact by the study team, and must serve pregnant women and/or women with young children. A sample of 30 health workers per upazilla were invited to participate in the survey using random exclusion of eligible workers in the roster. A national total of 600 surveys (300 in intervention areas, 300 in comparison areas) were completed in 2017.

### 2.4. Variable definition

A summary of all variables included in the analysis is presented in Table 1.

**Table 1:** Summary of variables included in analysis.

Variable type	Variable name	Definition	Measurement time points
Primary outcome	IYCF service delivery	Continuous (0–14); count of topics covered by the health worker during IYCF counseling (self-reported; range 0–14) (endline: no recall period specified; post-endline: in the last 30 days)	Endline and post-endline
Intermediate outcomes	IYCF knowledge	Continuous (0–14); count of breastfeeding and complementary feeding knowledge items answered correctly	Baseline, endline, and post-endline
	Job satisfaction	Binary; very satisfied vs. not very satisfied	Baseline, endline, and post-endline
	Job readiness	Binary; health worker felt they had adequate training for their job	Baseline, endline, and post-endline
Effect modifiers (program activities)	Comprehensiveness of refresher training	Continuous (0–1); proportion of self-reported topics a health worker recalled receiving at their last refresher training	Endline and post-endline
	Receipt of incentives	Binary; health worker reported receipt of incentives in past year	Endline and post-endline
	Incentive amount	Continuous; amount of incentive received in past year	Endline and post-endline
Covariates	Cadre	Binary; standard health workers vs. higher-level health workers	Baseline, endline, and post-endline
	Age	Continuous; in years	Baseline, endline, and post-endline
	Time spent in job	Continuous; in years	Baseline, endline, and post-endline
	Education level	Categorical; none, some or completed primary, some or completed secondary, and higher than secondary	Baseline, endline, and post-endline

The primary outcome for this analysis was quality of IYCF service delivery, defined as the count of topics covered by the health worker during IYCF counseling (self-reported; range 0–14); this measure is available at endline and post-endline. Eligible IYCF topics that health workers could mention are listed in Supplementary Appendix Table S1.

We also assessed three intermediate outcomes hypothesized to be on the causal pathway between the A&T intervention and service delivery (Figure 1). First, we defined IYCF knowledge (both breastfeeding and complementary feeding) as the count of items answered correctly during the baseline, endline, and post-endline surveys (range 0–14). Items included were common across all three surveys (Supplementary Appendix Table S1). Second, we defined job satisfaction as a binary variable (very satisfied vs. not very satisfied). At baseline and endline, this was measured using a 10-point scale and “very satisfied” was operationalized as scores ≥8 on this scale; at post-endline, this was measured using a 5-point scale, and “very satisfied” was defined as reporting ≥4. Third, job readiness was a binary variable representing whether the health worker felt they had adequate training for their job (binary yes/no variable).

We hypothesized that two A&T intervention activities could modify sustainment of effects: (1) comprehensiveness of refresher training, defined as the proportion of self-reported topics a health worker recalled receiving at their last refresher training (measured only at endline and post-endline); and (2) receipt of incentives, measured through two variables: a binary variable representing whether the health worker received incentives in the last year, and a continuous variable representing the amount of incentive received in the last year (with 0 if no incentive was received). Both financial incentive variables were measured only at endline and post-endline.

We adjusted for a number of covariates that could impact IYCF service delivery and its sustainment over time, including heath worker cadre (binary: standard health workers defined as Shasthya Shebika [SS]/Pushti Shebika [PS], vs. higher-level health workers including Shasthya Kormi [SK]/Pushti Kormi [PK]), age in years (continuous), years in job (continuous), and education level (categorical: none, some or completed primary, some or completed secondary, and higher than secondary).

### 2.5. Statistical analysis

We specified multivariable linear regression models (for continuous outcomes, including quality of service delivery, knowledge score, and incentive amount), and linear probability models (for binary outcomes, including job satisfaction, job readiness, and whether the health worker received an incentive in the previous 12 months). Linear probability models were utilized for binary outcomes to aid in ease of interpretation of findings. Difference-in-difference (DID) estimates were generated by interacting an indicator variable representing intervention areas (vs. comparison areas) and an indicator variable representing time (baseline, endline, and post-endline, with endline as the reference time point). To assess effect modification by refresher training quality and financial incentives, we included three-way interaction terms (intervention vs. comparison, and the hypothesized modifier), with lower order (two-way) interactions and main effects included. To visualize the three-way interaction terms, we dichotomized the refresher training variable using the 10th and 90th percentile of topics covered during training. For each model, we generated marginal predicted probabilities of the outcome at each time point in intervention and comparison areas. These marginal predicted probabilities allowed us to compare between-group differences (intervention vs. control) at each time point. Furthermore, we assessed whether the slope differed between each time point for intervention and control. Within-group changes in the post-endline period should be interpreted as exploratory. All models included robust standard errors allowing for intragroup correlation clustered at the upazilla (intervention) level. Models were adjusted for health worker cadre, age, years in job, and education level. Analyses were carried out in Stata v17.

## 3. Results

A total of *n* = 290 health workers were surveyed at baseline (147 in intervention areas, 143 in comparison areas), *n* = 511 at endline (347 in intervention areas, 164 in comparison areas), and *n* = 600 at post-endline (300 in each area) (Table 2). Most respondents were SS/PS cadre and had an average number of 5–10 years in their current role. The sample was not fully balanced at endline and post-endline: respondents in comparison areas had more years of experience, and educational attainment was different; respondents from the comparison were also slightly older at endline and there was a difference in the percentage of cadre represented at post-endline (Supplementary Appendix Table S2).

**Table 2:** Sample characteristics among frontline health workers at each survey round.

	Baseline survey, 2010 (*n* = 290)		Endline survey, 2014 (*n* = 511)		Post-endline survey, 2017 (*n* = 600)	
	Intervention areas (*n* = 147)	Comparison areas (*n* = 143)	Intervention areas (*n* = 347)	Comparison areas (*n* = 164)	Intervention areas (*n* = 300)	Comparison areas (*n* = 300)
Cadre, % (*n*)						
SK/PK	27.2 (40)	33.6 (48)	43.2 (150)	36.0 (59)	39.0 (117)	22.0 (66)
SS/PS	72.8 (107)	66.4 (95)	56.8 (197)	64.0 (105)	61.0 (183)	78.0 (234)
Years spent in role, mean (SD)	6.0 (4.4)	5.3 (4.0)	4.8 (4.6)	7.6 (5.1)	7.6 (5.0)	9.9 (6.0)
Age, mean (SD)	36.6 (10.5)	34.9 (10.4)	35.4 (10.6)	38.9 (12.4)	39.9 (10.8)	40.3 (10.9)
Years of schooling, % (*n*)						
None	27.2 (40)	18.9 (27)	11.5 (40)	18.3 (30)	10.0 (30)	8.0 (24)
Primary, some or completed	23.8 (35)	27.3 (39)	30.6 (106)	22.6 (37)	30.7 (92)	29.3 (88)
Secondary, some or completed	44.2 (65)	47.6 (68)	42.4 (147)	51.8 (85)	38.0 (114)	53.0 (159)
Beyond secondary	4.8 (7)	6.3 (9)	15.6 (54)	7.3 (12)	21.3 (64)	9.7 (29)

### 3.1. Sustainment of program activities and outcomes at each round

#### 3.1.1. Primary outcome

**Figure 2:**
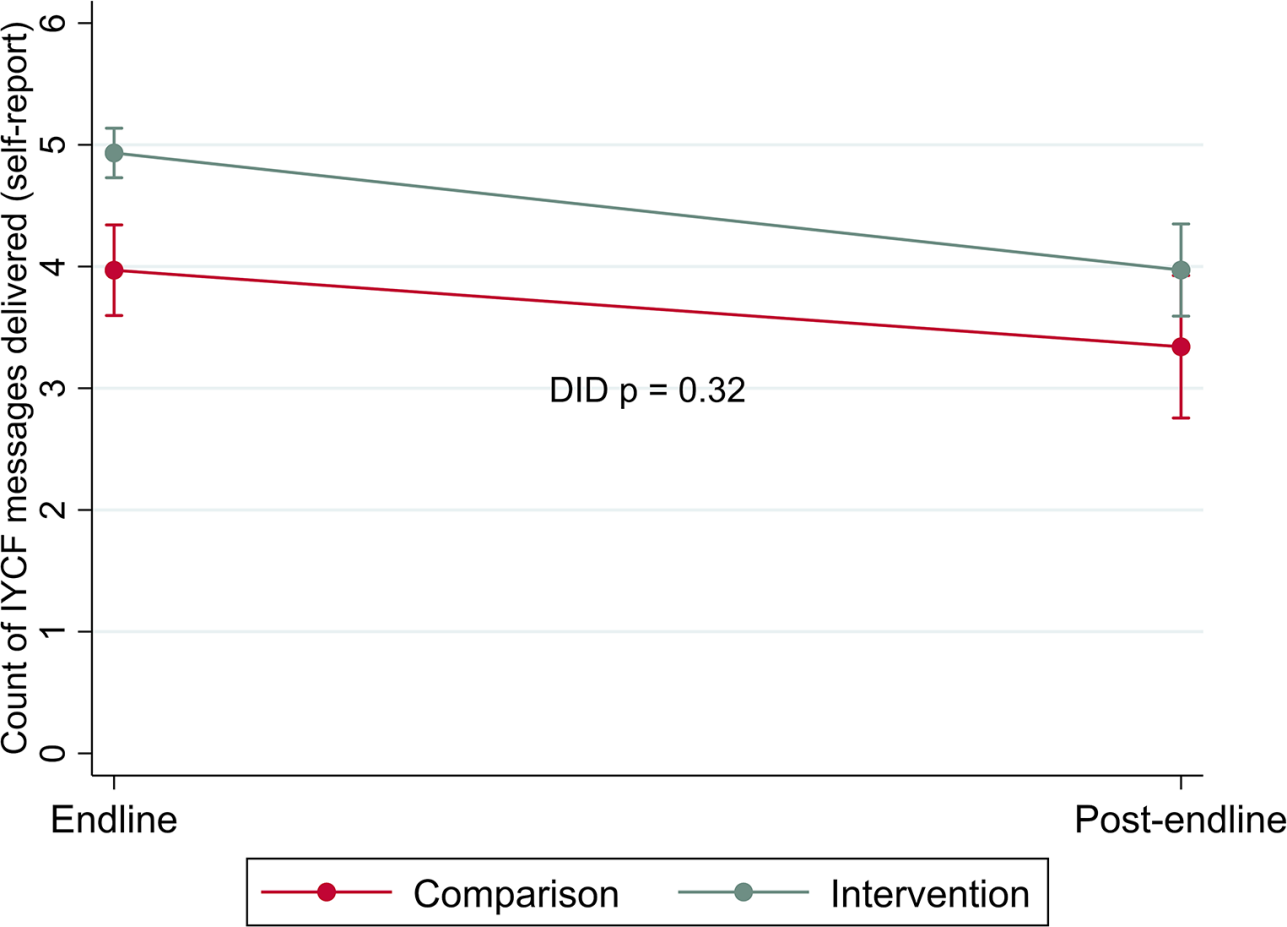
Changes in delivery of the quantity of IYCF messages included during counseling by area over time (difference in difference estimates).

The program effect was not sustained for quality of IYCF service delivery, i.e., number of IYCF topics discussed during care. As shown in Table 3 (comparing the outcome at each time point for intervention vs. control) and Figure 2 (demonstrating the differences in the changes in the outcome over time), the adjusted marginal predictions of self-reported number of IYCF topics discussed during IYCF counseling visits was significantly higher among health workers in intervention areas than comparison areas at endline (4.93 [SE 0.10] topics covered by health workers in intervention areas and 3.97 [SE 0.18] topics covered by health workers in comparison areas). By post-endline, delivery of IYCF messages declined among both intervention and comparison health workers, with no significant difference in this decline (DID *p* = 0.32). The difference between intervention and comparison areas was marginally significant at post-endline (3.97 [SE 0.18] topics covered by health workers in intervention areas and 3.34 [SE 0.28] topics covered by health workers in comparison areas).

**Table 3:** Primary and intermediate outcomes and program activities at each survey round: marginal predicted outcomes resulting from difference-in-difference models (SE in parentheses); *p*-values for the null hypothesis of no difference in predicted outcomes in intervention and comparison areas at each round.

	Baseline survey, 2010			Endline survey, 2014			Post-endline survey, 2017		
	Intervention areas	Comparison areas	*p*-value	Intervention areas	Comparison areas	*p*-value	Intervention areas	Comparison areas	*p*-value
Primary outcome									
Count of IYCF messages delivered (range 0–14)	n/a	n/a	n/a	4.93 (0.10)	3.97 (0.18)	<0.001	3.97 (0.18)	3.34 (0.28)	0.067
Intermediate outcomes									
Total IYCF knowledge score (range 0–14)	9.51 (0.64)	9.59 (0.26)	0.91	12.80 (0.12)	11.09 (0.082)	<0.001	12.65 (0.95)	11.13 (0.050)	<0.001
Satisfied with job (binary 0–1)	0.69 (0.063)	0.60 (0.060)	0.28	0.79 (0.062)	0.55 (0.058)	0.0079	0.48 (0.044)	0.38 (0.038)	0.10
Job readiness (binary 0–1)	0.79 (0.060)	0.83 (0.045)	0.58	0.96 (0.013)	0.86 (0.032)	0.0021	0.83 (0.029)	0.86 (0.019)	0.28
Program activities									
Proportion of topics covered at last refresher training (range 0–1)	n/a	n/a	n/a	0.38 (0.017)	0.044 (0.017)	<0.001	0.15 (0.0070)	0.13 (0.0092)	0.018
Received incentive in last 12 months (binary 0–1)	n/a	n/a	n/a	0.90 (0.03)	0.47 (0.077)	<0.001	0.054 (0.017)	0.11 (0.021)	0.046
Amount of incentive received in last 12 months (continuous)	n/a	n/a	n/a	530.90 (34.92)	101.25 (38.86)	<0.001	22.14 (6.84)	30.64 (8.15)	0.38

Adjusted for cadre, years spent in role, age, and education. Standard errors are clustered at the upazilla level.

#### 3.1.2. Intermediate outcomes

**Figure 3:**
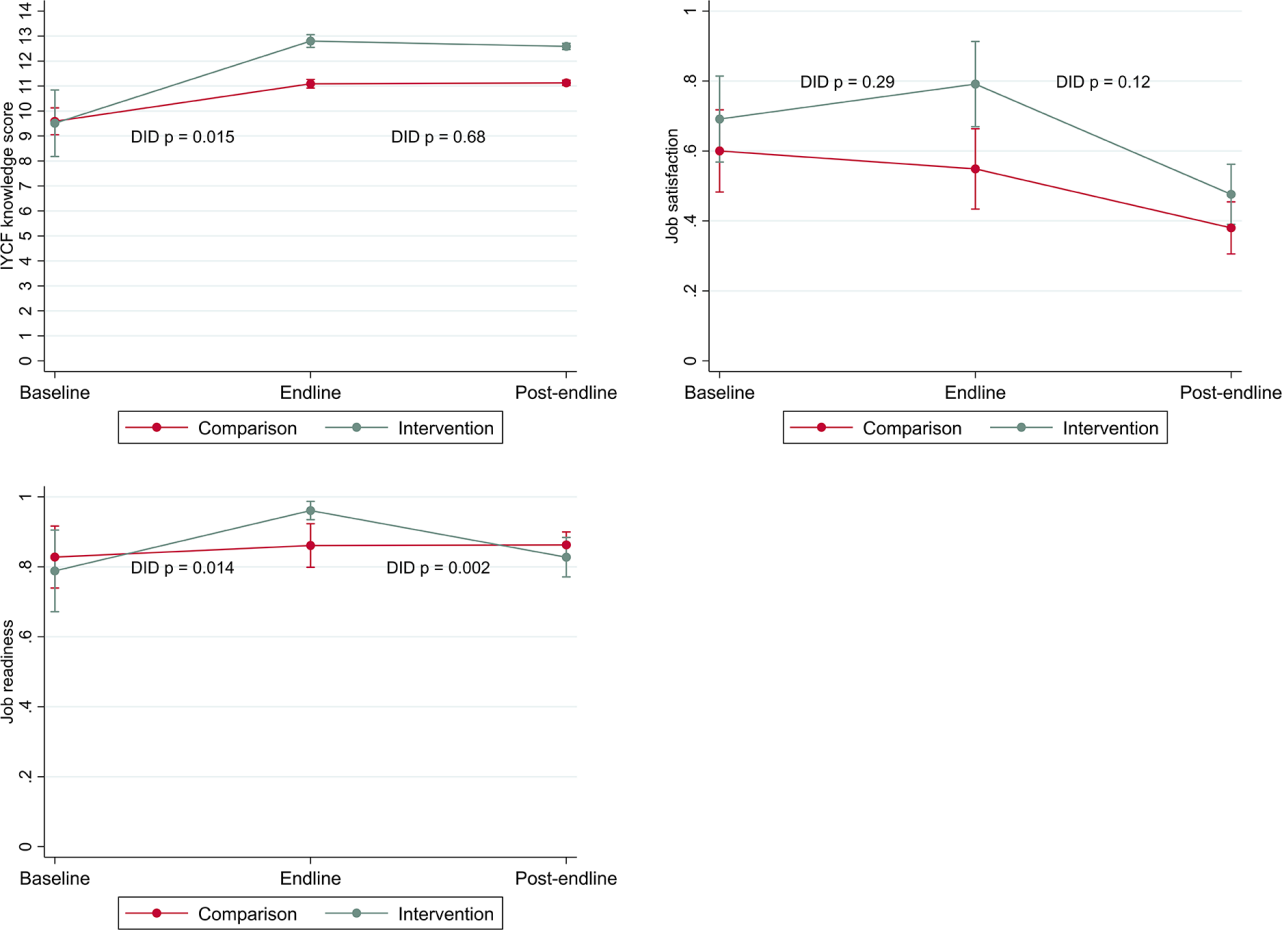
Changes in intermediate outcomes (IYCF knowledge, job satisfaction, job readiness) by area over time (difference in difference estimates).

Findings for intermediate outcomes over time are presented in Table 3 (comparing the outcome at each time point for intervention vs. comparison) and Figure 3 (demonstrating differences in changes in the intermediates outcomes over time). At baseline, there were no substantive differences between all intermediate outcomes—IYCF knowledge, job satisfaction, and job readiness—comparing intervention and comparison areas. At endline, significantly better IYCF knowledge was observed among all health workers in both intervention and comparison areas compared to baseline, but gains were significantly larger among health workers in intervention areas. There was evidence of a sustained program effect for IYCF knowledge as these improvements in intervention areas persisted to post-endline. The gap between intervention and comparison areas remained largely unchanged by post-endline (IYCF knowledge score of 12.65 [SE 0.95] among intervention health workers vs. 11.13 [SE 0.050] among comparison workers), with no detectable difference in the slope for these changes between endline and post-endline.

In comparison areas, job satisfaction declined across all periods. In intervention areas, job satisfaction increased during the project period (baseline to endline); however, there was no detectable difference in the changes over time (DID *p* = 0.29). Subsequently, job satisfaction among workers in intervention areas declined at post-endline and reached a similar level as comparison areas (48% [SE 4.4] satisfied health workers in intervention areas and 38% [SE 3.8] satisfied health workers in comparison areas).

In intervention areas, health workers experienced an increase in reported job readiness by endline, while there was no change among workers in comparison areas (DID *p* = 0.014). However, by post-endline intervention-area workers had returned to their baseline level, while health workers in comparison areas reported no change (DID *p* = 0.002).

#### 3.1.3. Program activities

**Figure 4:**
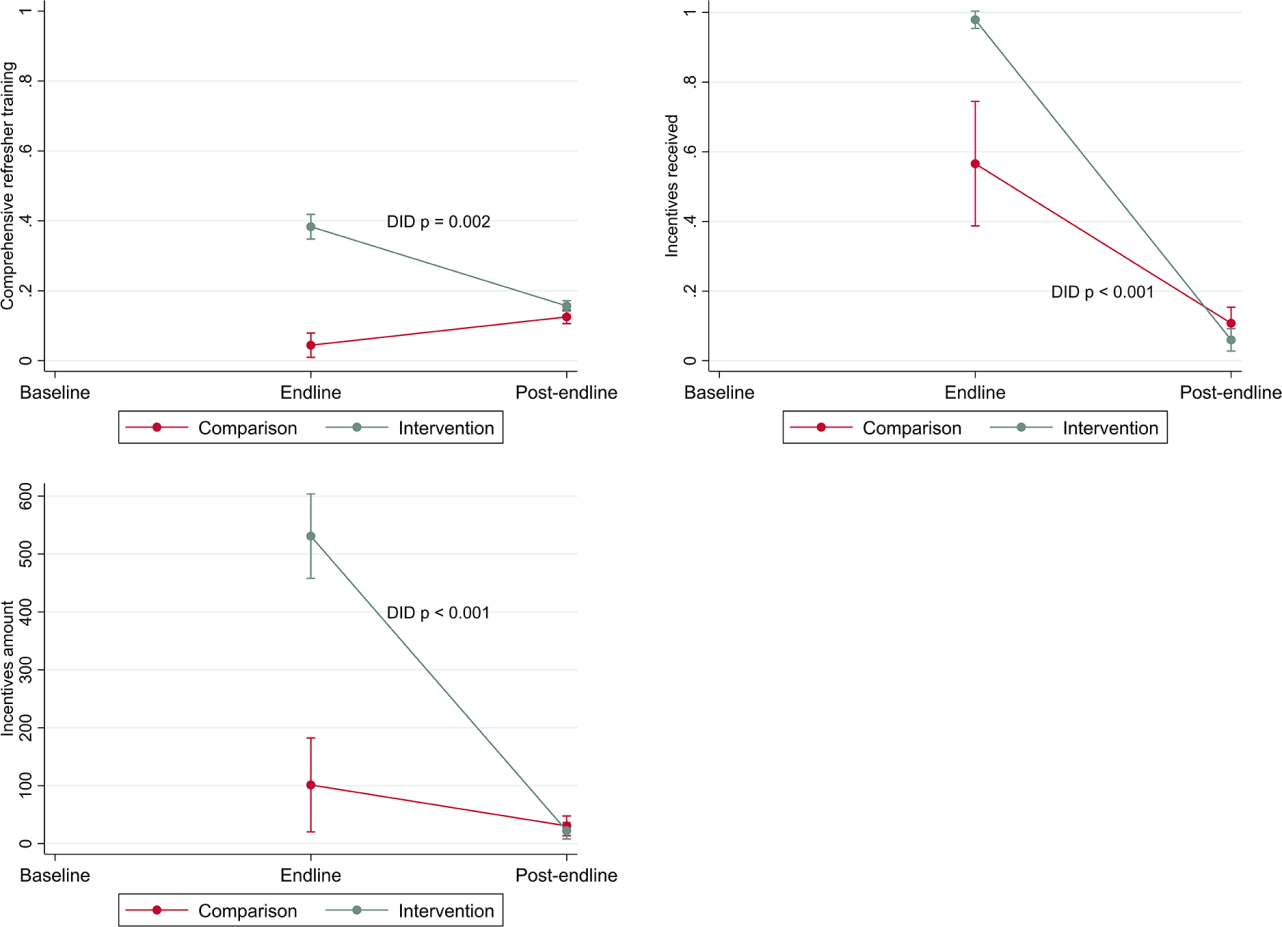
Changes in program activities (refresher trainings, incentives received, incentive amount) by area over time (difference in difference estimates).

Sustainment of program activities are presented in Table 3 (comparing the outcome at each time point for intervention vs. comparison) and Figure 4 (demonstrating differences in changes in the program activities over time). Despite declining from endline to post-endline (DID *p* = 0.002), IYCF topics covered at last refresher training remained significantly higher in intervention areas at post-endline vs. comparison areas. Incentive payments declined substantially from endline to post-endline among both intervention and comparison health workers, with a steeper decline among those in intervention areas (DID *p* < 0.001). At endline, incentive payments were significantly more common in intervention areas: 90% of workers in intervention areas and 46% of workers in comparison areas said they had received an incentive payment. At post-endline, these values had fallen to 5% and 11%, respectively.

### 3.2. Factors modifying the sustainment of program effects

**Figure 5:**
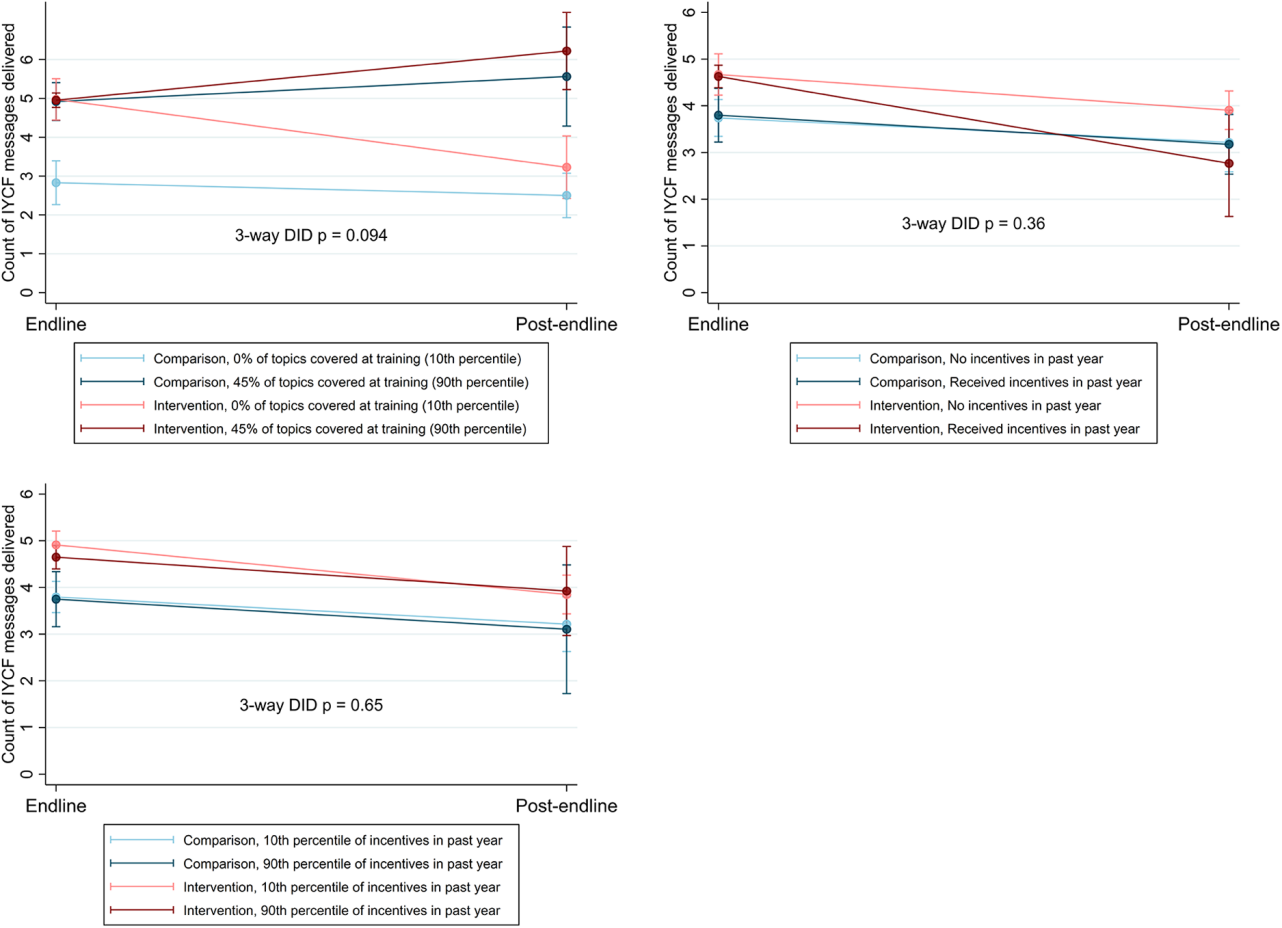
Changes in delivery of IYCF messages during counseling by area over time, including interaction terms for program activities.

We assessed whether program activities modified the sustainment of program effects of the primary outcome (IYCF service delivery, Figure 5).

Comprehensive refresher trainings potentially protected against the deterioration of the primary outcome (service delivery): workers who reported more comprehensive refresher trainings (above the 90th percentile of reported topics discussed) delivered significantly more IYCF messages during counseling by post-endline, in both intervention and comparison areas. Workers in intervention areas who had less-comprehensive refresher trainings experienced the largest declines in IYCF counseling by post-endline. However, more comprehensive IYCF refresher training was not associated with any difference in knowledge (intermediate outcome) across treatment groups, between endline and post-endline (Supplementary Appendix Figure S1). There was however no apparent interaction between comprehensiveness of refresher training, and job satisfaction or job readiness, in either group over time (Supplementary Appendix Figures S2, S3).

There was no apparent effect of incentives, whether presence of any incentive or the amount of incentive, on quality of IYCF service delivery nor on intermediate outcomes (Figure 5 and Supplementary Appendix Figures S1–S3), with the exception of job readiness—health workers in intervention areas with no incentive reported lower job readiness at endline, but converged with their peers at post-endline.

## 4. Discussion

This paper adds to the empirical literature on sustainability as there are relatively few quantitative studies that estimate the degree to which program outcomes endure over time (19). We find mixed evidence about the sustainment of outcomes from the Alive & Thrive initiative three years after the program's conclusion in Bangladesh. On the main outcome of IYCF service delivery and quality (i.e., number of topics discussed during counseling), we see evidence of “voltage drop” (28): the intervention was associated with a significant improvement in service delivery at endline, but three years later, quality had declined for workers in both intervention and comparison areas and was no longer significantly different between the groups.

Importantly, we find that although there were not enduring improvements in intervention areas for the main outcome of service quality, a critical intermediate outcome—IYCF knowledge—was sustained, as was an associated related program activity of IYCF topics covered at refresher trainings. This indicates that interventions that seek to improve health worker knowledge may have an enduring effect but that this may not continue to impact behaviors. This adds a new dimension to the “know-do gap” literature, which has demonstrated the potential disconnect between what health workers know and their clinical practice (35–39). Prior IYCF research in Ethiopia found mothers with better access to nutrition education had higher knowledge scores, improved child feeding practices, and reduced rates of stunting among their children (40). However, while knowledge may be a necessary ingredient to achieving—and sustaining—behavior change, it may not be sufficient. Future research should assess health workers' knowledge-sharing efficacy and other factors that may also impact IYCF service delivery and quality (41).

Two key intermediate outcomes—job satisfaction and job readiness—increased over the program period but this improvement was not sustained. Our previous research has indicated that this may be directly attributable to the end of A&T: removal of incentives has been shown to negatively impact BRAC health workers' IYCF service delivery quality (42) and desire to perform (43).

Alive & Thrive was designed as a “proof of concept” initiative: if it demonstrated success, then governments would have the evidence necessary to implement it. Although some activities have endured in Bangladesh, not all have continued in the format or intensity as in the initial design (14). Externally-funded programs and projects cannot continue forever; these findings add new insights to the growing literature on sustainability by measuring sustainment across a program's theory of change. If, as in this case, an intervention can have lasting effects on health worker knowledge, but cannot continue to make material contributions that may help translate this knowledge into action—for example, incentive payments or job readiness—what are reasonable sustainability expectations? It is important to consider this finding in the context of the study population: BRAC community health workers include both paid and unpaid (volunteers), and different types of health workers may differentially respond to programs, and to their end (44, 45). Further, there may be other factors—including macroeconomic factors or structural changes in the healthcare system—that may impact intermediate outcomes, like job satisfaction for health workers. Previous research has explored how social factors, like religious norms, may affect BRAC workers' job performance (46). It is important to consider contextual factors like these when preparing for and assessing sustainability.

This is also particularly noteworthy when considering our exploratory finding that refresher trainings may protect against the “voltage drop” in service quality. More research that attempts to disentangle sustainment of effects across a program's theory of change may help policymakers prioritize areas for continued investment after programs end, in order to catalyze ongoing impacts.

This analysis is innovative in its design and approach, but is not without limitations. First, the primary outcome—number of IYCF topics discussed during recent counseling visits—is not a perfect measure of service quality. It was self-reported and blinding of the intervention was impossible so health workers in intervention areas so may have over-reported their performance. Additionally, IYCF counseling is not a “one-size-fits-all” activity and the topics discussed will naturally differ across clients, so more topics is not necessarily a measure of high-quality counseling. There were also measurement challenges with this variable owing to different recall periods: no recall period was specified at endline, but at post-endline health workers were only asked about counseling in the last 30 days. This may account for at least some of the decline in messages reportedly delivered. Second, some outcomes were measured only at endline and post-endline, so we cannot assess whether the observed declines represent returns to baseline levels. Third, response options varied slightly between survey rounds; for example, the job satisfaction variable used 10-point Likert scale in the baseline and endline surveys, but a 5-point scale in the post-endline survey. This may account for some difference in responses over time. Fourth, this analysis was not powered to detect changes over time, nor to assess effect modification over time. Nevertheless, the findings do indicate evidence of “voltage drop,” and suggest some factors that may protect against declines in program sustainment. To assess whether the hypothesized effect modifiers could instead be mediators, we conducted a mediation analysis using Barron and Kenney methods to test whether the difference-in-difference coefficient was statistically different with and without potential mediators in the model—however the results showed no statistical difference. Fifth, this analysis focused solely on outcomes among health workers and did not include key IYCF measures among caregivers of children, such as exclusive breastfeeding; although this is a limitation, our previous research has found that service quality (IYCF messages delivered during counseling) is associated with improved IYCF outcomes in this population (33). Lastly, some outcomes may have been impacted by social desirability bias (such as job satisfaction and job readiness); however, we do not expect this bias to differ between intervention and comparison areas nor over time.

This was a unique analysis: we linked three separate cross-sectional surveys—performed over a 7-year period and spanning intervention baseline, endline and post-endline—to quantitatively examine sustainment of program outcomes and activities. The study teams deliberately aligned survey tools and sampling frames in order to enable this analysis, and both the study design and its limitations offer useful lessons for scholars of sustainability. We find evidence of “voltage drop” in the primary outcome of quality of service delivery, although refresher trainings may protect against this deterioration. There are sustained improvements in knowledge among health workers in intervention areas compared to their counterparts in comparison areas, but this was not sufficient to achieve sustained outcomes. We hope this analysis stimulates more research to empirically measure and quantify sustainment, particularly across interventions' theories of change and into post-endline periods.

## Data Availability

The datasets presented in this study can be found in online repositories. The names of the repository/repositories and accession number(s) can be found below: https://dataverse.harvard.edu/dataverse/AliveandThrive.
