# Supplementary material for: Assessing sustainment of health worker outcomes beyond program end: Evaluation results from an infant and young child feeding intervention in Bangladesh
Source: Front Health Serv. 2023 Jan 9;2:1005986. doi: 10.3389/frhs.2022.1005986 (PMC10012630; doi:10.3389/frhs.2022.1005986)
Supplement: Supplementary file 1 [file Datasheet1.docx]

Appendix Table 1: Outputs and outcomes at each survey round in intervention and comparison areas

|  | 2010 | | 2014 | | 2017 | |
| --- | --- | --- | --- | --- | --- | --- |
| *Variable* | Intervention  N=147 | Comparison  N=143 | Intervention  N=347 | Comparison  N= 164 | Intervention  N=300 | Comparison  N=300 |
| **Program Activities** |  |  |  |  |  |  |
| **REFRESHER TRAININGS** |  |  |  |  |  |  |
| Proportion of topics covered at last refresher training (range 0-1), mean (SD) |  |  | 0.38 (0.22) | 0.07 (0.12) | 0.15 (0.08) | 0.13 (0.09) |
| Received incentive in last 12 months |  |  | 99% | 55% | 4% | 11% |
| Amount of incentive received in last 12 months (continuous) mean (SD) |  |  | 536 (300) | 99 (268) | 16 (113) | 32 (114) |
| **Intermediate Outcomes** |  |  |  |  |  |  |
| **KNOWLEDGE** |  |  |  |  |  |  |
| Baby should be breastfed immediately | 91.8 (125) | 94.4 (135) | 100 (347) | 97.0 (159) | 99.0 (297) | 96.7 (290) |
| Give baby colostrum | 97.3 (14) | 97.0 (97.9) | 95.6 (335) | 98.8 (162) | 100 (300) | 99.7 (299) |
| Baby should be given expressed breastmilk when mother is away | 31.3 (46) | 11.2 (16) | 96.3 (334) | 36.0 (59) | 92.7 (278) | 36.7 (110) |
| HW knows >1 reason to exclusively breastfeed | 70.8 (104) | 87.4 (125) | 100 (347) | 100 (164) | 99.3 (298) | 97.0 (291) |
| Baby <6 m should not be given water in hot weather | 51.1 (71) | 38.7 (48) | 97.4 (338) | 74.4 (122) | 97.7 (293) | 83.7 (251) |
| Continue BF until child is 24 mo old | 95.2 (140) | 94.4 (135) | 98.6 (342) | 95.1 (156) | 97.3 (292) | 95.7 (287) |
| Continue BF even if the mother is pregnant | 23.8 (35) | 36.4 (52) | 84.4 (293) | 82.9 (136) | 91.3 (274) | 83.0 (249) |
| Most common reason for sore/painful breasts is poor positioning and attachment | 23.8 (35) | 17.5 (25) | 42.1 (146) | 17.7 (29) | 24.3 (73) | 13.8 (41) |
| Mother should breastfeed more often/frequently if she thinks her child is not getting enough breastmilk | 23.8 (35) | 20.1 (30) | 79.8 (277) | 26.2 (43) | 80.3 (241) | 35.0 (105) |
| Only breastmilk until 6 months | 77.6 (114) | 81.1 (116) | 99.4 (345) | 97.0 (159) | 99.3 (298) | 96.0 (288) |
| Total BF knowledge score (0-10), mean (SD) | 6.1 (1.8) | 6.0 (1.4) | 9.0 (0.8) | 7.2 (1.2) | 8.8 (0.9) | 7.4 (1.3) |
| Meal frequency 6-9 months | 99.2 (124) | 99.2 (132) | 100 (347) | 99.4 (163) | 99.7 (299) | 99.7 (299) |
| Meal frequency 9-12 months | 99.2 (122) | 98.5 (158) | 95.1 (330) | 92.7 (152) | 98.7 (296) | 97.7 (293) |
| Meal frequency 12-14 months | 99.2 (119) | 98.5 (127) | 99.1 (344) | 98.2 (161) | 100 (300) | 99.7 (299) |
| Responsive feedings | 85.7 (126) | 83.9 (120) | 96.8 (335) | 92.7 (152) | 85.0 (255) | 73.7 (221) |
| Total CF knowledge score (0-4), mean (SD) | 3.3 (1.2) | 3.5 (1.0) | 3.9 (0.3) | 3.8 (0.4) | 3.8 (0.4) | 3.7 (0.5) |
| Total IYCF knowledge score (range 0-14), mean (SD) | 9.4 (2.5) | 9.5 (1.9) | 12.9 (0.9) | 11.1 (1.3) | 12.6 (1.0) | 11.1 (1.4) |
| **JOB SATISFACTION** |  |  |  |  |  |  |
| Satisfied with job (binary 0-1), % (n) | 66.7 (98) | 58.0 (83) | 78.5 (274) | 51.5 (85) | 49.3 (148) | 40.0 (120) |
| **JOB READINESS** |  |  |  |  |  |  |
| Job readiness (binary 0-1), % (n) | 76.9 (113) | 81.8 (117) | 95.9 (237) | 83.6 (138) | 84.0 (252) | 87.7 (263) |
| **Primary Outcome** |  |  |  |  |  |  |
| **MESSAGES DELIVERED** |  |  |  |  |  |  |
| Initiate BF immediately after birth |  |  | 60.6 (180) | 59.1 (62) | 39.8 (105) | 37.6 (99) |
| Observe and ensure EBF after birth up to 6 months of age |  |  | 87.5 (260) | 81.0 (85) | 70.8 (187) | 63.5 (167) |
| Demonstrate and observe position and attachment for BF to mothers |  |  | 43.4 (129) | 6.7 (7) | 56.8 (150) | 25.5 (67) |
| Identify and solve problems related to EBF |  |  | 17.5 (52) | 1.0 (1) | 9.9 (26) | 9.1 (24) |
| At 5 months’ visit, discuss with mothers/family members about feeding their baby family food in addition to breast milk when the baby completes six months |  |  | 17.5 (52) | 20.1 (22) | 22.4 (59) | 21.3 (56) |
| Feeding during illness of the child (increase frequency and duration of breastfeeds) |  |  | 8.8 (26) | 2.9 (3) | 4.9 (13) | 3.0 (8) |
| Frequency and quantity of complementary food |  |  | 69.7 (207) | 48.6 (51) | 21.6 (57) | 25.1 (66) |
| Demonstrate how to prepare baby’s food from family food |  |  | 22.9 (68) | 16.2 (17) | 33.7 (89) | 24.7 (65) |
| Counsel mothers about continuous BF for 2 years |  |  | 59.6 (177) | 42.9 (45) | 26.5 (70) | 24.0 (63) |
| Identify and solve problems related to BF and feeding family food |  |  | 31.7 (94) | 12.4 (13) | 17.4 (46) | 9.9 (26) |
| Discuss feeding during and after illness of child (increase breastfeeding, more and frequent feeding of family foods after illness for 10 days to 2 weeks,, food that the child likes) |  |  | 20.9 (62) | 1.9 (2) | 6.1 (16) | 3.0 (8) |
| Hand washing with soap |  |  | 36.0 (107) | 19.1 (20) | 59.5 (157) | 40.7 (107) |
| Pushtikona |  |  | 10.1 (30) | 15.2 (16) | 42.1 (111) | 41.8 (110) |
| How to maintain good appetite in the child (offer different varieties, wait till child is hungry, do not force feed, do not fill up stomach with water/liquids/junk foods, praise the child for eating, encourage self-feeding, take time to feed patiently) |  |  | 11.5 (34) | 5.7 (6) | 63.6 (168) | 43.7 (11.5) |
| Count of messages delivered (range 0-14), mean (SD) |  |  | 5.0 (1.7) | 3.3 (1.3) | 4.2 (2.4) | 3.3 (2.3) |

|  | **Baseline survey** | | | **Endline survey** | | | **Post-endline survey** | | | |
| --- | --- | --- | --- | --- | --- | --- | --- | --- | --- | --- |
|  | Intervention (n=147) | Comparison (n=143) | p-value | Intervention (n=347) | Comparison (n=164) | p-value | Intervention (n=300) | Comparison (n=300) | p-value |  |
| Cadre, % (n) |  |  |  |  |  |  |  |  |  |  |
| SK/PK | 27.2 (40) | 33.6 (48) | 0.24 | 43.2 (150) | 36.0 (59) | 0.12 | 39.0 (117) | 22.0 (66) | <0.001 |  |
| SS/PS | 72.8 (107) | 66.4 (95) |  | 56.8 (197) | 64.0 (105) |  | 61.0 (183) | 78.0 (234) |  |  |
| Years spent in role, mean (SD) | 6.0 (4.4) | 5.3 (4.0) | 0.15 | 4.8 (4.6) | 7.6 (5.1) | <0.001 | 7.6 (5.0) | 9.9 (6.0) | <0.001 |  |
| Age, mean (SD) | 36.6 (10.5) | 34.9 (10.4) | 0.16 | 35.4 (10.6) | 38.9 (12.4) | 0.001 | 39.9 (10.8) | 40.3 (10.9) | 0.69 |  |
| Years of schooling, % (n) |  |  |  |  |  |  |  |  |  |  |
| None | 27.2 (40) | 18.9 (27) | 0.50 | 11.5 (40) | 18.3 (30) | 0.01 | 10.0 (30) | 8.0 (24) | <0.001 |  |
| Primary, incomplete | 10.2 (15) | 15.4 (22) |  | 18.2 (63) | 14.0 (23) |  | 18.3 (55) | 16.3 (49) |  |  |
| Primary, complete | 13.6 (20) | 11.9 (17) |  | 12.4 (43) | 8.5 (14) |  | 12.3 (37) | 13.0 (39) |  |  |
| Secondary, incomplete | 19.1 (28) | 21.0 (30) |  | 14.7 (51) | 20.7 (34) |  | 17.0 (51) | 37.0 (111) |  |  |
| Secondary, complete | 25.2 (37) | 26.6 (38) |  | 27.7 (96) | 31.1 (51) |  | 21.0 (63) | 16.0 (48) |  |  |
| Beyond secondary | 4.8 (7) | 6.3 (9) |  | 15.6 (54) | 7.3 (12) |  | 21.3 (64) | 9.7 (29) |  |  |

Appendix Table 2: Sample characteristics at each survey round with p-values for null hypothesis significance test of difference between intervention and comparison at each survey wave.

|  |  |
| --- | --- |
|  |  |

**Appendix Figure 1:** Changes in IYCF knowledge score by area over time, including interaction terms for program outputs.

|  |  |
| --- | --- |
|  |  |

**Appendix Figure 2:** Changes in job satisfaction by area over time, including interaction terms for program outputs.

|  |  |
| --- | --- |
|  |  |

**Appendix Figure 3:** Changes in job readiness by area over time, including interaction terms for program outputs.
